# A Treatise on Physiology and Hygiene for Educational Institutions and General Readers

**Published:** 1884-11

**Authors:** 


**A Treatise on Physiology and Hygiene for Educational Institutions and General Readers**. By JOSEPH C. HUTCHINSON, M. D., LL. D. Fully illustrated. New York: Clark & Maynard, Publishers, 734 Broadway. 1884.

A work on physiology and hygiene, prepared by a competent writer, for the use of schools, has long been desired, and we believe the present work fills this want. It is sufficiently comprehensive in its scope, and simple in its details to meet the purpose for which it was written. The language used is familiar, technical terms being avoided, as far as possible. It is well illustrated. It has a chapter on the use of the microscope in the study of physiology, with an appendix devoted to the resuscitation of the drowned, care of sick rooms, etc. We think the author and publishers have performed a duty to the public in furnishing this work, which we cordially endorse and commend.

